# Oral Hygiene for Children

**Published:** 1913-04-15

**Authors:** William Lyon

**Affiliations:** Jackson, Mich.


### ORAL HYGIENE FOR CHILDREN.

WILLIAM LYON, M.D., JACKSON, MICH.

It seems presumptuous indeed for a mere doctor of medicine to speak of oral hygiene to doctors of Dental Surgery, but as I have been given the opportunity I shall endeavor to present to you some of the problems as observed in the routine examination of school children. This work has enabled us to form a very clear idea of the splendid results which come from adequate dental care and the deplorable and needless results of dental neglect.

The thorough digestion of proper food being of prime importance in the development of all children, and the oral cavity being the first division of the digestive tract it has an importance that is often underestimated and probably never overrated.

As dentists you ought to consider yourselves Division Superintendents on the main line of the road to Healthtown and work in accordance with the responsibility of your high office.

The child with decayed teeth very often can do no thorough mastication, and because of this may develop more or less serious digestive disturbance, which never fails to make its evil impress upon development. Furthermore I am sure that food, no matter how wholesome it may be, in many mouths that I have seen must receive so great an admixture of filth that it is a splendid example of vital resistance that the child survives the oft repeated infections. Many children show an entire lack of appetite for breakfast which can be readily accounted for by the intense aroma of the rotting material which has festered and accumulated over night, thus he misses a meal which he needs to sustain him during his morning of activity and at noon he will likely overeat—with poor mastication—and repeat the same thing at evening. Hence he can scarcely escape some impairment of development in the face of such a handicap.

There is at least occasionally a physician who contends himself with prescribing at some condition which his patient may name, thus avoiding the bother of making a careful study of the patient and the application of proper treatment. However the best men are those who really examine their patients and who lean strongly toward preventive medicine, and in their treatment of children aim at a good foundation for adult life. The functions of the field of dentistry may seem small and unimportant but the child's nourishment is almost wholly dependent on good mastication. All dentists should be teachers of oral hygiene and never fail to use every means necessary to the full conservation of all the teeth in childhood as they are relatively so much more important than for adults. We recently saw a child of twelve who went to an eminently respectable dentist to have a tooth extracted because it ached, and this was accomplished with neatness and dispatch, but no mention was made to the child concerning three permanent teeth with cavities and urgent need of a thorough dental cleaning, so it was left to us to make recommendation in the matter. I also encountered a child not seven years old with decayed deciduous molars and very poor oral hygiene, who returned a slip upon which the attending dentist had written "Nature is doing all she can for this child." We can easily agree with him, but we are convinced that he failed to do what any wide-a-wake dentist could have done easily to improve upon the rank botch that nature was making of the job.

I believe it to be the rule and not the exception for physicians to ignore oral hygiene in dealing with children, and I have known many instances of physicians extracting permanent teeth which could surely have been saved if referred to competent dentists for treatment. The Doctor of Dental Surgery being a specialist in the strictest sense of that term, should be consulted even more frequently than we consult specialists in some other lines for the benefit of our patients, because the physician has not had training that would supply him even the rudiments of dental science. We have only to observe the beautiful results of the work of the dental surgeons in fractures of the jaw to emphasize this point. And there is that classical example of the need of co-operation in those cases where the nose and throat specialist must remove the underlying cause of arch deformity and kindred ills before the dentist can accomplish anything like the good results he so much desires. The development and durability of the permanent teeth is so dependent upon good general nutrition; and good general nutrition is so thoroughly dependent upon good oral hygiene in early childhood that I feel that the neglect of the teeth of these little ones is a sin of omission so grave that it should not easily be forgiven. Of course I realize that if everything possible in the way of maintenance of oral hygiene is carried out from early childhood that there will ultimately be less bridge and plate work to do, but you will have to use so much time in this matter of conservation that your time for that sort of patchwork will be limited as it ought to be.

Certainly all dentists and physicians should actively co-operate in this matter of teaching and establishing good oral hygiene and realize that the very best for each child is none too good. With intelligent medical and dental co-operation and proper home and school environment there should result a marked improvement in general good health and physical development of the coming generation. The medical profession is beginning to recognize that prevention is the ideal of its future and it will be the part of wisdom for the dental profession also to give more attention to this phase of its obligation to the public.

